# Synergism of non-thermal plasma and low concentration RSL3 triggers ferroptosis via promoting xCT lysosomal degradation through ROS/AMPK/mTOR axis in lung cancer cells

**DOI:** 10.1186/s12964-023-01382-z

**Published:** 2024-02-12

**Authors:** Shengjie Peng, Guodong Chen, K. N. Yu, Yue Feng, Lele Zhao, Miaomiao Yang, Wei Cao, Waleed Abdelbagi Ahmed Almahi, Mingyu Sun, Yuan Xu, Ye Zhao, Cheng Cheng, Fengqin Zhu, Wei Han

**Affiliations:** 1 Anhui Province Key Laboratory of Medical Physics and Technology, Institute of Health and Medical Technology, Hefei Institutes of Physical Science, Chinese Academy of Sciences, Hefei, 230031 People’s Republic of China; 2 University of Science and Technology of China, Hefei, 230026 People’s Republic of China; 3 Hefei Cancer Hospital, Chinese Academy of Sciences, Hefei, 230031 People’s Republic of China; 4 Department of Physics, City University of Hong Kong, Tat Chee Avenue, Kowloon Tong, Hong Kong, People’s Republic of China; 5 State Key Laboratory in Marine Pollution, City University of Hong Kong, Tat Chee Avenue, Kowloon Tong, Hong Kong, People’s Republic of China; 6 Teaching and Research Section of Nuclear Medicine, School of Basic Medical Sciences, Anhui Medical University, Hefei, 230032 People’s Republic of China; 7 Institute of Plasma Physics, Hefei Institutes of Physical Science, Chinese Academy of Sciences, Hefei, 230031 People’s Republic of China; 8 Collaborative Innovation Center of Radiation Medicine of Jiangsu Higher Education Institutions and School for Radiological and Interdisciplinary Sciences (RAD-X), Soochow University, Suzhou, 215006 People’s Republic of China

**Keywords:** Non-thermal plasma, Ferroptosis, Lung cancer, RSL3 clinical application

## Abstract

**Background:**

Though (1S, 3R)-RSL3 has been used widely in basic research as a small molecular inducer of ferroptosis, the toxicity on normal cells and poor pharmacokinetic properties of RSL3 limited its clinical application. Here, we investigated the synergism of non-thermal plasma (NTP) and low-concentration RSL3 and attempted to rise the sensitivity of NSCLC cells on RSL3.

**Methods:**

CCK-8 assay was employed to detect the change of cell viability. Microscopy and flowcytometry were applied to identify lipid peroxidation, cell death and reactive oxygen species (ROS) level respectively. The molecular mechanism was inspected with western blot and RT-qPCR. A xenograft mice model was adopted to investigate the effect of NTP and RSL3.

**Results:**

We found the synergism of NTP and low-concentration RSL3 triggered severe mitochondria damage, more cell death and rapid ferroptosis occurrence in vitro and in vivo. NTP and RSL3 synergistically induced xCT lysosomal degradation through ROS/AMPK/mTOR signaling. Furthermore, we revealed mitochondrial ROS was the main executor for ferroptosis induced by the combined treatment.

**Conclusion:**

Our research shows NTP treatment promoted the toxic effect of RSL3 by inducing more ferroptosis rapidly and provided possibility of RSL3 clinical application.

**Graphical Abstract:**

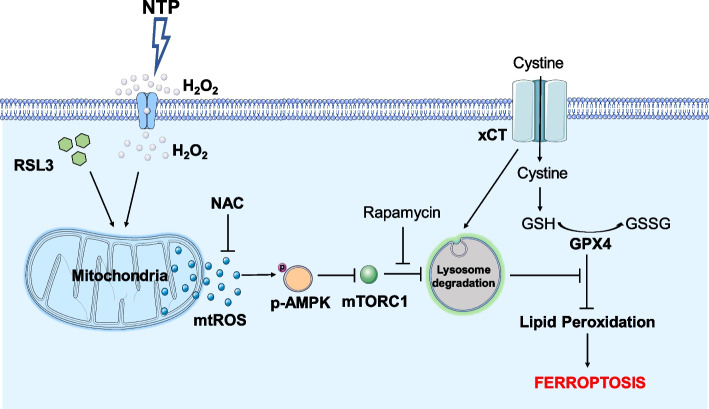

Video Abstract

**Supplementary Information:**

The online version contains supplementary material available at 10.1186/s12964-023-01382-z.

## Summary

Ferroptosis, a novel form of programmed cell death driven by cellular oxidative metabolism and iron-dependent lipid peroxidation, was recently implicated in kinds of diseases, including cancer therapy. Herein, we found the synergism of non-thermal plasma (NTP) and low-concentration RSL3 triggered mitochondria damage and ferroptosis rapidly in non-small-cell lung cancer (NSCLC) cells. Mechanistically, NTP and RSL3 synergistically induced not only the increase of reactive oxygen species (ROS) but also xCT lysosomal degradation through ROS/AMPK/mTOR signaling, resulting in excessive lipid peroxidation and ferroptosis. Besides, our study showed that ROS generated in mitochondria was the main executioner for ferroptosis induced by the combined treatment. Our research shows the potential clinical availability of RSL3 and the feasibility of combining NTP with clinical therapeutic strategies in cancer treatment.

## Background

Non-thermal plasma, also called NTP, is an ionized gaseous substance composed of reactive species, electrons, ions, neutral particles, and ultraviolet photons, etc. [1, 2]. To date, extensive studies indicate the potential application of NTP in cancer therapy and NTP treatment has shown its notable anti-cancer capacity in vitro and in vivo [3, 4]. Mechanistically, NTP treatment increases the intracellular ROS level, causes oxidative stress, damages macromolecules, and ultimately induces cell death. Abundant reports have verified that NTP provokes DNA lesion through ROS and reactive nitrogen species (RNS) [5, 6], or induces mitochondria damage via undermining the mitochondrial membrane potential and Ca^2+^ accumulation [7, 8]. Moreover, previous studies have revealed that NTP induces apoptosis [9–11], autophagy [12, 13], pyroptosis [14] and senescence [15] in tumor cells, and these programmed cell death pathways triggered by NTP are being increasingly recognized in oncology therapy.

Ferroptosis, a newly discovered form of regulated cell death, is characterized by iron-dependent and accumulation of lipid peroxidation, leading to disordered cellular redox homeostasis and finally cell death [16]. xCT is an important membrane antiporter and takes charge of exporting intracellular glutamate in exchange for extracellular cystine to promote GSH synthesis, which maintains cellular redox homeostasis and protects cells against ferroptosis. Numerous studies demonstrate that mTORC1, AMPK, p53, BAP1 and some other regulators exhibit significant roles in modulating xCT expression and regulating ferroptosis [17]. In recent years, ferroptosis has been proved to be involved in tumor therapy, especially in the clearance of drug-resistant tumor cells. Among those chemicals inducing ferroptosis, (1S, 3R)-RSL3, a GPX4 inhibitor, is frequently applied in vitro. However, many studies have reported that the doses of RSL3, which effectively induced tumor cell death, showed significant cytotoxicity on normal cells including nephrocyte [18], neuron [19], fibroblast [20], etc. Furthermore, poor pharmacokinetic properties of RSL3 have been revealed in animal studies [21–23]. All those disadvantages have restricted the potential clinical application of RSL3.

Recently, NTP has been identified to promote the release of ferrous ion in ferritin [24]. Oral squamous cell carcinoma cells were killed by NTP treatment in an iron-dependent manner [25], and plasma-activated Ringer’s lactate was found to switch cell death from autophagy to ferroptosis by lysosomal nitric oxide [26]. Herein, we combined NTP with RSL3 and found that low doses of NTP significantly promoted ferroptosis rapidly in tumor cells treated with RSL3 at low concentrations, which had no distinct toxicity in vitro and in vivo. Furthermore, the activated signaling axis, ROS/AMPK/mTOR, and the facilitated xCT lysosomal degradation were discovered. These results supported that NTP could synergize with RSL3 in “shoving” cancer cells across the thresholds of ferroptosis, augment the killing effect in vitro and suppressing tumor growth in vivo. The present study could provide hints to boosting the pharmacological application of RSL3 in tumor therapy and to further understanding of cell death induced by NTP.

## Materials and methods

### Cell culture, reagents, and antibodies

Human lung cancer cell lines, Calu-1 and H1299 were purchased from American Type Culture Collection (Manassas, USA), while H1975, HCC827, A549 and PC9 cells were obtained from the Cell Bank of Type Culture Collection of Chinese Academy of Sciences (Shanghai, China). Calu-1, H1975, HCC827 and H1299 cells were cultured in RPMI 1640 medium (Hyclone, Logan, USA) supplemented with 10% fetal bovine serum (FBS, Thermo Scientific Hyclone, Logan, USA) and 1% penicillin/streptomycin (Gibco, Carlsbad, CA, USA). Other cells were cultured in high glucose Dulbecco’s modified Eagle medium (DMEM, Hyclone, Logan, USA) supplemented with 10% FBS and 1% penicillin/streptomycin. All cells were maintained in a humidified incubator with 5% CO_2_ at 37 °C.

Caspase inhibitor (Z-VAD-FMK, Z-VAD) and necrosis inhibitor (Necrosis-1 s, Nec-1 s) were purchased from Selleck Chemicals (Houston, TX, USA). Autophagy inhibitor (Chloroquine, CQ), GPX4 inhibitor (RSL3), and ferroptosis inhibitor (Ferrostatin-1, Fer-1) were purchased from Sigma-Aldrich (New York, USA). CCK-8 Assay Kit and N-acetyl-L-cysteine (NAC) were purchased from Beyotime Biotechnology (Shanghai, China). Puromycin was obtained from Gibco (Life Technologies, Carlsbad, CA, USA). The primary antibodies against GPX4 (ZEN-BIO, Cat#381958), xCT (Cell Signaling Technology, Cat#12691), AMPK (Cell Signaling Technology, Cat#5831S), phospho-AMPK (Cell Signaling Technology, Cat#50081S), mTOR (Cell Signaling Technology, Cat#2983S), phospho-mTOR (ZEN-BIO, Cat#381557), P70S6K (Cell Signaling Technology, Cat#9202S), phospho-P70S6K (Cell Signaling Technology, Cat#9234S), β-actin (HuaBio, Cat#EM21002) were used in immunoblotting. The secondary IRDye-labeled goat anti-mouse and anti-rabbit IgG antibodies were purchased from LI-COR Biosciences (LI-COR, Lincoln, NE, USA).

### NTP equipment and treatment

Atmospheric pressure dielectric barrier discharge (DBD) plasma was used in this study [14], [27]. The NTP generator consisted of a hollow plexiglass cylinder as the reaction chamber with four electrodes and two orifices as gas inlet and gas outlet. Each high voltage electrode was a 32-mm-diameter copper column covered with 1-mm-thick quartz glass as an insulating dielectric barrier. Another four 32-mm-diameter copper columns were set as grounding electrodes. The NTP was generated by the voltage of 12 kV (peak to peak) with the frequency of 24 kHz. The discharge power density was detected to be about 0.9 W/cm^3^. The discharge gap between the bottom of quartz glass and the surface of the medium was maintained at 5 mm. Helium gas (99.999%) was used as the working gas with a flow rate of 120 L/h. Cells were seeded in 35-mm-diameter petri dishes with 1.5 mL complete medium. For NTP treatment, cells were exposed to NTP for a preset time, which determined the dose of NTP treatment. Plasma-activated medium (PAM) was made from the basic medium exposed directly to NTP. For combined treatments, the cells were pretreated with RSL3 for 4 h, and then treated with NTP.

### Immunoblotting

At 24 h after treatment, the cells were lysed with RIPA buffer supplemented with phosphatase inhibitor, PMSF (Beyotime Biotechnology, Shanghai, China), and were then sonicated. The protein concentration was determined with BCA Protein Assay Reagent Kit (Beyotime Biotechnology, Shanghai, China). Approximately 40 μg of each protein sample was separated by SDS-PAGE, then transferred onto PVDF membranes (Millipore Corporation, Bedford, MA, USA). The membranes were blocked with 5% defatted milk for 1 h at room temperature and then incubated with primary antibodies overnight at 4 °C. After washing three times with TBST (0.1% Tween-20 in Tris-HCl buffer), the membranes were incubated with the fluorescent secondary antibodies (Alex Fluor 790 goat anti-rabbit IgG,1:10,000; Alex Fluor 680 goat anti-mouse IgG, 1:10,000) for 1 h at room temperature. Membranes were imaged with a LI-COR Odyssey Imaging System and protein levels were quantified with ImageJ software.

### Quantitative RT-PCR

Total RNA was extracted from cells with HiPure Total RNA Mini Kit (Magen, Shanghai, China) following the manufacturer’s instructions. cDNA was synthesized with ProtoScript First Strand cDNA Synthesis Kit (Yeasen, Shanghai, China). Quantitative PCR reaction mixtures were prepared with Hieff qPCR SYBR Green Master Mix (Yeasen, Shanghai, China). PCR reactions were performed and analyzed on LightCycler 480 Instrument (Roche, Indianapolis, IN). PCR primers were shown as follows: 5′-TCTCCAAAGGAGGTTACCTGC-3′, 5′-AGACTCCCCTCAGTAAAGTGAC-3′ (xCT); 5′-CGGAACCGCTCATTGCC-3′, 5′-ACCCACACTGTGCCCATCTA-3′ (β-actin). Relative mRNA levels were normalized to β-actin mRNA level. Triplicate samples per condition were analyzed and each measurement was performed for three times.

### Cell viability assay

At 24 h after treatment, the cell viability was determined with CCK-8 Assay Kit following the manufacturer’s instructions. The absorbance was measured at 450 nm with a Varioskan Flash microplate reader (Thermo Fisher Scientific, Rockford, IL, USA).

### Generation of stable xCT overexpression cell lines

Human xCT overexpression plasmids (Miaoling Bio, Wuhan, China) were packaged into lentivirus particles. Virus-containing supernatant was collected at 48 h after transfection and the infected Calu-1 cells were seeded in 6-well dishes. At 72 h after viral infection, cells were selected with puromycin (0.75 μg/mL) for 7 to 10 days and then tested for xCT expression by western blotting analysis.

### Cell death analysis with flow cytometry

At 24 h, the treated cells were harvested and stained with Annexin V-FITC and PI (BD Bedford, MA, USA) for 15 min at room temperature. The cells were then immediately analyzed with a flow cytometer (Accuri C6, BD Biosciences, Bedford, MA, USA). All the data analysis was performed with Flowjo analysis software (TreeStar, Ashland, OR, USA).

### Detection of lipid peroxidation

For imaging, the cells were washed with PBS, stained with C11 BODIPY 581/591 (5 μM) for 30 min, and then photographed with a fluorescent microscope (Leica DMI 4000B, Germany) at 12 h after treatment. All images were collected with the same instrument parameters and processed with the same settings.

For flow cytometry analysis, after being incubated with C11 BODIPY 581/591 (5 μM), the cells were trypsinized and collected gently, filtered into single-cell suspensions, and immediately analyzed with a flow cytometer (Accuri C6, BD Biosciences, Bedford, MA, USA). A minimum of 10,000 cells were analyzed for each sample, and each experiment was independently performed three times and representative experimental results are shown. All the data analyses were performed with Flowjo analysis software (TreeStar, Ashland, OR, USA).

### MitoROS measurement

Mitochondrial ROS was measured with the fluorescent probe MitoSOX Red (Invitrogen, Grand Island, NY, USA). After 12 h, the treated cells were washed with PBS, incubated with the probe for 30 min and then quantified with the flow cytometry.

### Detection of H_2_O_2_ level

The H_2_O_2_ level was determined with the Hydrogen Peroxide Assay Kit (Beyotime Biotechnology, Shanghai, China). The medium treated with NTP was harvested immediately and mixed with the detecting buffer. The absorbance of the supernatant was measured at 560 nm with the Varioskan Flash microplate reader.

### GPX4 activity assay

The activity of GPX4 was determined with Total Glutathione Peroxidase Assay Kit (Beyotime Biotechnology, Shanghai, China). At 12 h, the treated cells were harvested and completely lysed with RIPA buffer, incubated with GPX4 working buffer for 15 min, and then detected at 340 nm with a Varioskan Flash microplate reader.

### Xenograft tumor models

Six-week-old male nude mice were obtained from GemPharmatech Company (Nanjing, China). Calu-1 cells were suspended in cold PBS and 5 × 10^6^ cells were injected subcutaneously into the right dorsal flank of nude mice. After 7 days, mice were randomly divided into four groups (6 mice/group): the control group, the RSL3 group, the PAM group and the PAM+RSL3 treatment group. The PAM was prepared by RPMI 1640 medium exposed to NTP for 5 min. Both RSL3 (100 mg/kg) and PAM were administered by intertumoral injection 3 times a week. The mice were sacrificed six weeks later. The tumor volume was calculated as volume = length × width^2^ × 1/2. All animal experiments have been approval by Hefei Institutes of Physical Science Experimental Animal Ethics Committee.

### Statistical analysis

Statistical differences were performed with GraphPad Prism 8.0 software (San Diego, CA, USA). Data represent the mean ± SD from three independent experiments. *P*-values were analyzed with Student’s *t*-test. In all comparisons, *p* < 0.05 was considered significant (n.s.: not significant; *: *p* < 0.05; **: *p* < 0.01; ***: *p* < 0.001).

## Results

### NTP enhanced the killing effect of RSL3 on NSCLC cells

**Fig. 1:**
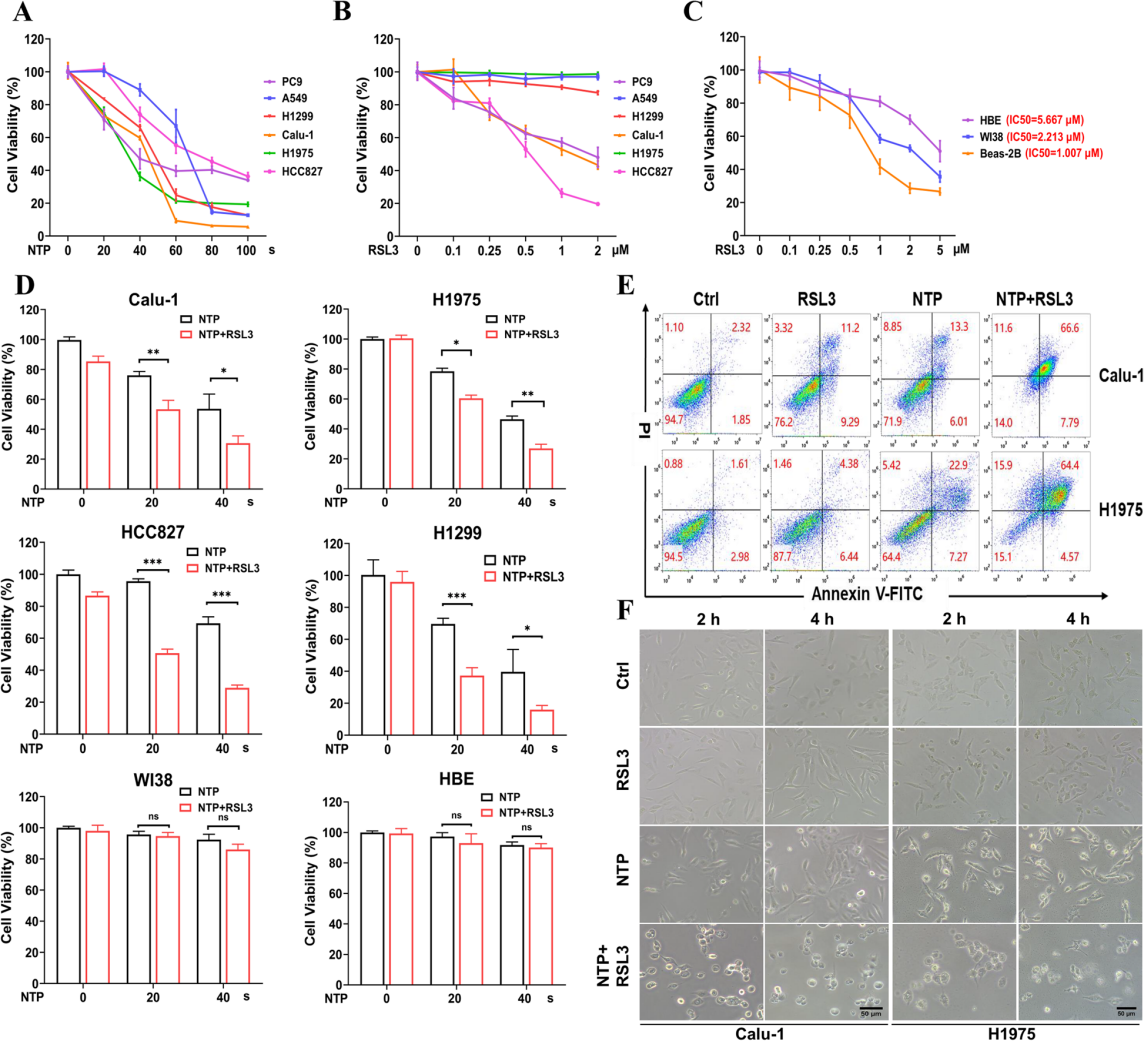
NTP enhanced the killing effect of RSL3 on NSCLC cells. Cell viability and cell death were measured at 24 h after indicated treatment. **A** Viability of NSCLC cells treated with NTP. B&C. Viability of NSCLC cells (**B**) and normal lung cells (**C**) treated with RSL3. **D** Viability of NSCLC cells and normal lung cells after NTP (20 s) + RSL3 (0.1 μM) treatment. **E** Flow cytometry of cell death analysis (Annexin V-FITC/PI). **F** Cell morphology captured at 2 and 4 h after treatment. Scale bar, 50 μm

To determine the sensitivity to RSL3 or NTP, NSCLC cell lines (PC9, A549, H1299, Calu-1, H1975 and HCC827) were treated with indicated doses of NTP (0 ~ 100 s) or indicated concentration of RSL3 (0 ~ 2 μM). Results in Fig. 1A showed that the cell viability decreased with increasing NTP dose. RSL3 also exhibited concentration-dependent cytotoxicity to the tested lung cancer cells. Meanwhile, the decrease in the viability of normal lung cells was also observed especially above 1 μM RSL3 (Fig. 1B, C).

To explore the possibility of NTP promoting RSL3-induced tumor cell death, we treated cells with NTP + RSL3. A low concentration (0.1 μM) of RSL3 was adopted, and no significant cytotoxicity was detected in tumor and normal cells after RSL3 treatment (Fig. 1D). Interestingly, distinctly decreased cell viability was observed in the NTP + RSL3 group when compared to NTP only (Fig. 1D). Furthermore, NTP + RSL3 treatment did not induce significant decrease in cell viability in normal lung cells (WI38 and HBE) (Fig. 1D). Cell death analysis also showed severe loss of cell membrane integrity, which revealed more cell death induced after NTP + RSL3 treatment (Fig. 1E). We also observed that the cell death induced by the combined treatment occurred rapidly (less than 4 h after treatment) based on morphological characteristics (Fig. 1F). In conclusion, NTP could selectively enhance the toxic effect of RSL3 on tumor cells.

### NTP + RSL3 treatment synergistically induced ferroptosis in NSCLC cells

**Fig. 2:**
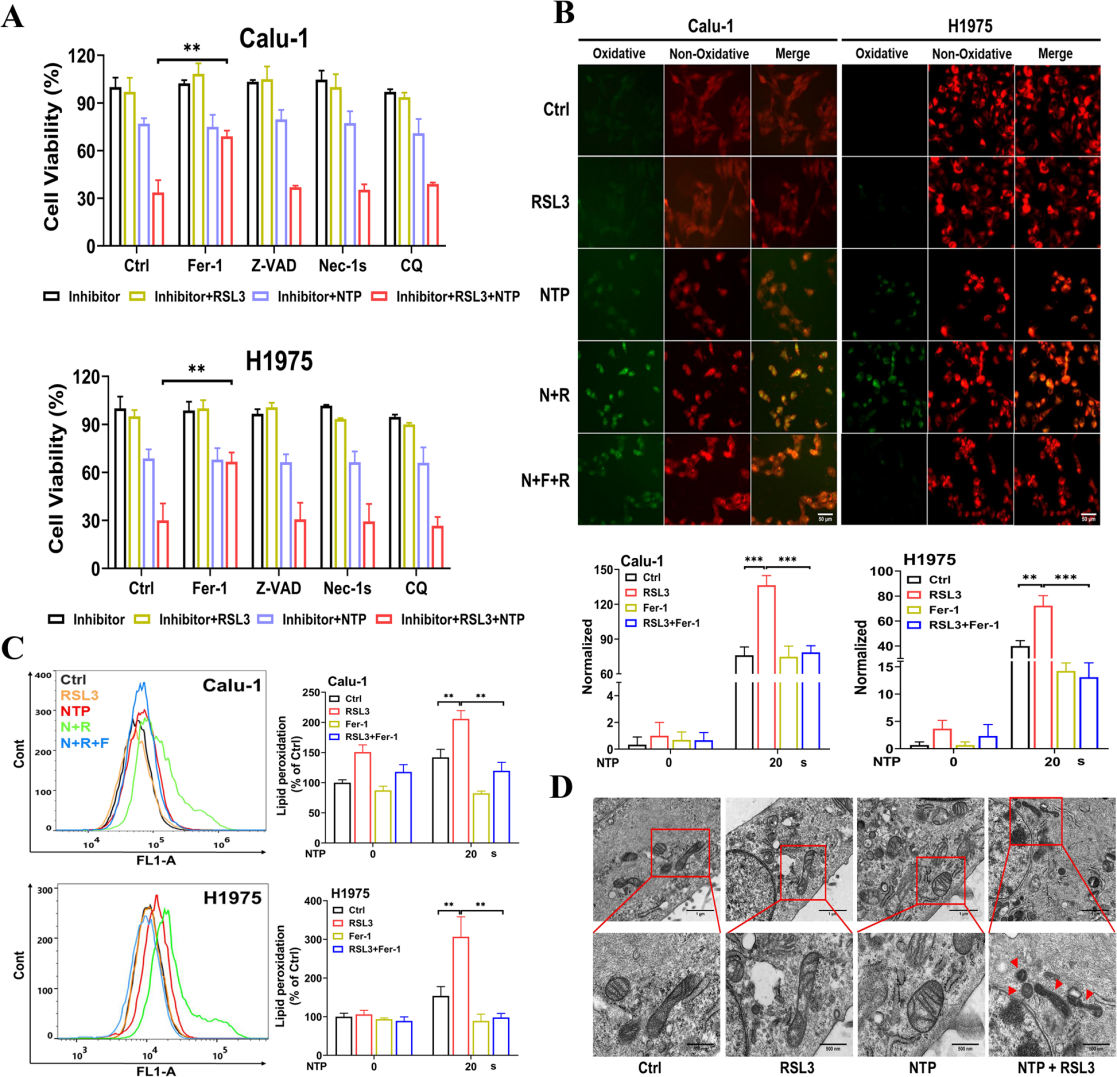
NTP + RSL3 treatment induces ferroptosis in NSCLC cells. **A** Effect of various inhibitors, Fer-1 (20 μM), Z-VAD-FMK (20 μM), Nec-1 s (20 μM) or CQ (100 μM) on cell viability after NTP + RSL3 treatment. **B** Typical fluorescent images and quantification of cellular lipid peroxidation at 24 h after treatment, N + R means NTP + RSL3, N + R + F means NTP + RSL3 + Fer-1. Scale bar, 50 μm. **C** Flow cytometry analysis of cellular lipid peroxidation. **D** Mitochondrial morphology captured by transmission electron microscopy. Scale bar, 1 μm (up) and 500 nm (down)

To clarify the type of cell death induced by combined treatment, different cell death inhibitors, Nec-1 s (necrosis inhibitor), Z-VAD-FMK (apoptosis inhibitor), Fer-1 (ferroptosis inhibitor) or CQ (autophagy inhibitor) were used. Results in Fig. 2A showed that only Fer-1 could significantly restore cell viability, indicating that combined treatment induced the occurrence of ferroptosis. Furthermore, the significantly increased accumulation of lipid peroxidation was also observed in Calu-1 and H1975 cells after combined treatment (Fig. 2B, C). Additionally, images captured by transmission electron microscopy also revealed ferroptosis-associated morphological changes, mitochondrial volume shrinkage, diminished or disappeared crista and increased mitochondrial membrane density after combined treatment, which were not observed after treatment with NTP or RSL3 alone (Fig. 2D). It was worth noting that only NTP (20 s) or RSL3 (0.1 μM) treatment did not induce ferroptosis but the combined treatment effectively induced ferroptosis. Our results confirm that NTP and RSL3 showed selective combined killing effect via inducing ferroptosis in NSCLC cells.

### NTP + RSL3 treatment synergistically generated mitochondrial ROS

**Fig. 3:**
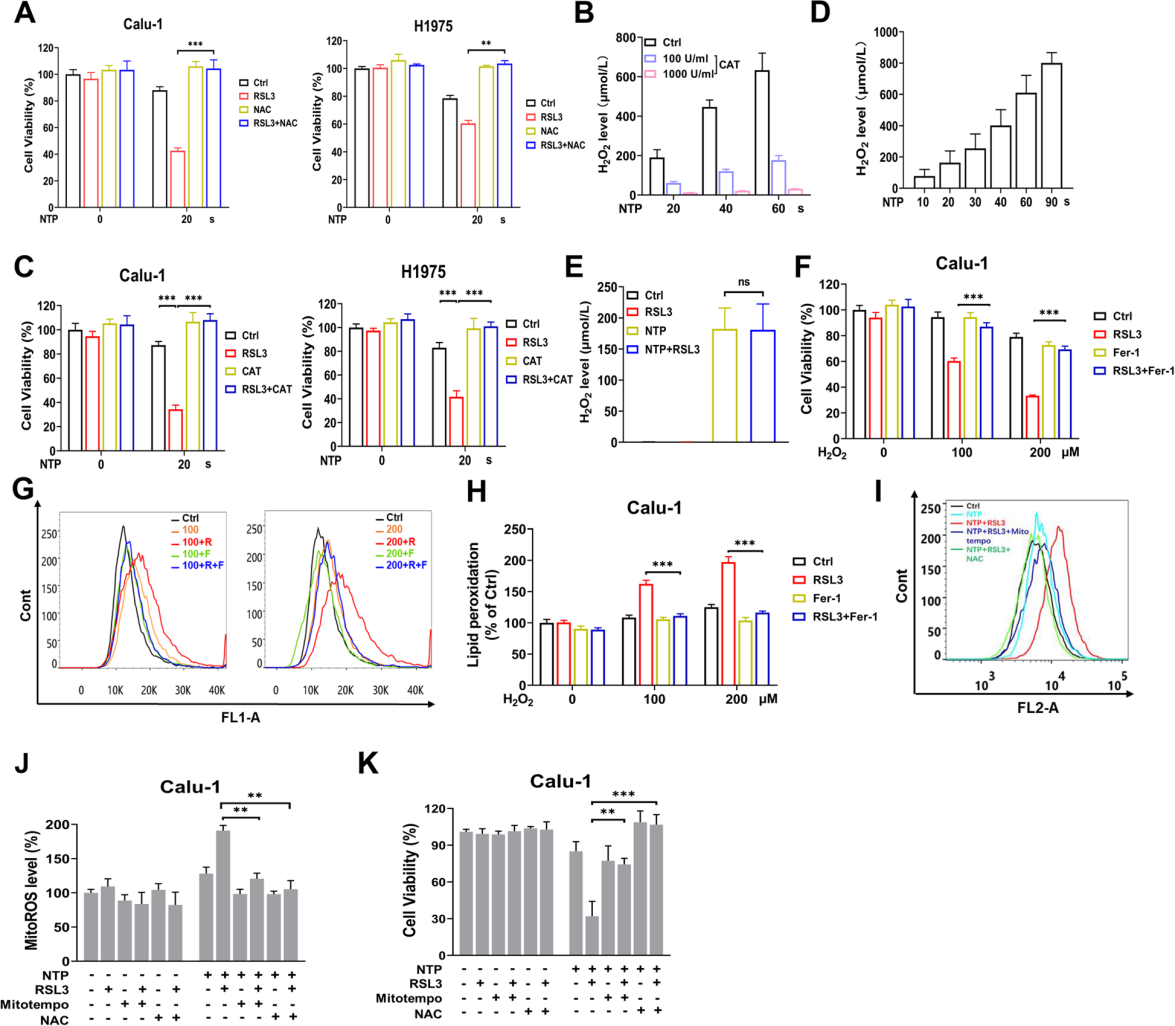
NTP + RSL3 treatment induces excessive mitochondrial ROS generation and ferroptosis. **A** Effect of NAC (5 mM) on cell viability treated with NTP + RSL3. **B** H_2_O_2_ level in PAM detected at 1 h after NTP treatment. **C** Effect of CAT (1000 U/ml) treatment on cell viability (24 h) treated with NTP + RSL3. **D** H_2_O_2_ level in the plasma-treated medium. **E** H_2_O_2_ level in medium containing RSL3 (0.1 μM) after NTP treatment. **F** Cell viability (24 h) after H_2_O_2_ + RSL3 treatment. **G** The lipid peroxidation level after H_2_O_2_ + RSL3 treatment. **H** Quantification of G. **I** The mitochondrial ROS level after NTP + RSL3 treatment. **J** Quantification of I. **K** Effect of Mitotempo (200 μM) or NAC (5 mM) on cell viability treated with NTP + RSL3

Considering that iron-mediated Fenton reaction leads to generation of free radicals and that accumulated lipid peroxidation and NTP is widely known to produce multiple kinds of ROS in the liquid phase [5], we hypothesized that NTP + RSL3 treatment would synergistically elevate intracellular oxidative stress and induce toxic effect. To prove this hypothesis, NAC was applied to scavenge the intracellular ROS, and effectively restored the viability of NTP + RSL3 treated NSCLC cells to the normal level, indicating that the killing effect of NTP + RSL3 was resulted from the synergistic ROS generation (Fig. 3A). As an essential kind of long-lived ROS in killing cancer cells by NTP reported in many previous studies, the effect of hydrogen peroxide (H_2_O_2_) was evaluated in NTP + RSL3 treatment. The catalase (CAT) treatment distinctly attenuated the H_2_O_2_ level in PAM and completely restored the decreased cell viability after NTP + RSL3 treatment to the control level (Fig. 3B, C). These results indicate that intracellular ROS generation played an important role in ferroptosis induced by the NTP + RSL3 treatment.

Why was ROS generation in NTP + RSL3 treated cells was more than that in cells treated only with NTP or RSL3? Considering it was well established that the NTP-originated reactive species in medium, especially H_2_O_2_, were transferred into cells via passive transportation with aquaporin and then resulted in further biological events [28, 29], we detected whether adding RSL3 into the medium (without cells) caused more H_2_O_2_ production than treating with NTP only. The results in Fig. 3D and E showed that no more H_2_O_2_ was produced after RSL3 addition, indicating that the excessive intercellular ROS after NTP + RSL3 treatment should be generated by the cells. Mimicking NTP with low concentration H_2_O_2_ also showed that H_2_O_2_ + RSL3 induced excessive decrease in the cell viability, and significantly increased the lipid peroxidation, which were not observed in cells treated with H_2_O_2_ or RSL3 only (Fig. 3F-H). These results show that the elevated oxidative stress and the occurrence of lethal effect in NTP + RSL3 treated cells were derived from the synergistic generation of intercellular ROS.

Given that mitochondria were the major source of intracellular ROS, we proposed that mitochondria-derived ROS might contribute to the elevated oxidative stress and play a key role in NTP + RSL3 treatment-induced ferroptosis. Surprisingly, the level of mitochondrial ROS, detected with MitoSOX Red, was increased significantly after NTP + RSL3 treatment. Furthermore, the increased mitochondrial ROS could be attenuated by MitoTempo, a specific mitochondrial ROS scavenger (Fig. 3I, J). Scavenging general ROS with NAC or scavenging mitochondrial ROS with MitoTEMPO showed the same effect of rescuing cell viability after NTP + RSL3 treatment, indicating that ROS originated from mitochondria mainly contributed to the elevated oxidative stress and ferroptosis (Fig. 3K).

Mechanistically, RSL3 restrained the enzyme activity via covalent binding and restricting GPX4 to catalyze hyperoxide into non-toxic alcohols [30, 31]. Previous studies have reported that mitochondria are highly sensitive to ferroptotic stimuli and displayed mitochondrial fragmentation and lipid peroxidation shortly after ferroptotic stimuli [32]. RSL3 treatment induced the increase of mitochondrial ROS, the loss of membrane depolarization, decrease of GSH and ultimately ferroptosis in cardiomyocytes [33]. The increased mitochondrial ROS in RSL3 treated group demonstrated that low concentration RSL3 had a potential effect on mitochondrial GPX4 activity and inhibited the enzyme catalyzing hyperoxide, which made cancer cells vulnerable to subsequent oxidative stress [34, 35]. In our work, the synergistic effect of NTP + RSL3 treatment induced the elevated level of mitochondrial ROS, which further activated ROS/AMPK/mTOR signaling pathway and promoted xCT lysosome degradation. We believed that the excessive mitochondrial ROS induced by NTP + RSL3 was the executor to cause xCT degradation and ferroptosis. Meanwhile, large amount of ROS induced by NTP was transferred into the cells [36], which were inadequate in eliminating hyperoxide species, resulting in excessive and lethal cellular oxidative stress and subsequent ferroptosis.

### NTP + RSL3 treatment synergistically inhibited xCT expression

**Fig. 4:**
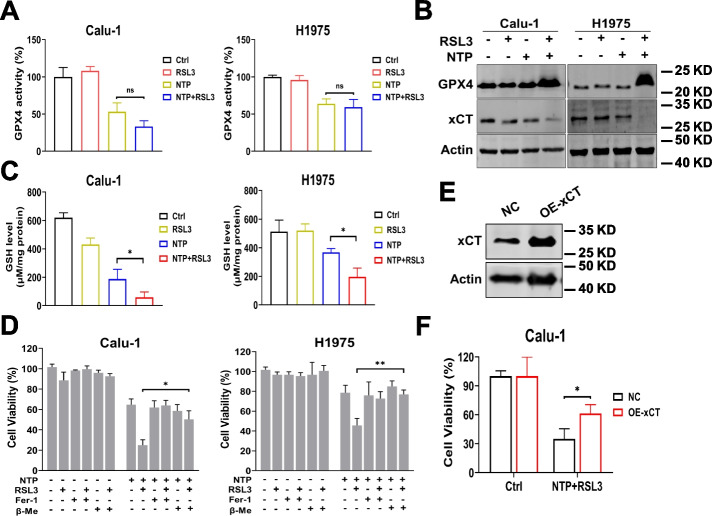
NTP + RSL3 treatment induces xCT dysfunction. **A** Activity of GPX4. **B** The protein expression of xCT and GPX4. **C** Level of GSH. **D** Effect of β-Me (100 μM) on the viability of cells treated with NTP + RSL3. **E** Overexpression of xCT in Calu-1 cells. **F** Effect of overexpression on viability of cells treated with NTP + RSL3

Given that RSL3 is a classical ferroptosis inducer via inhibiting GPX4 activity, we hypothesized NTP + RSL3 might first synergistically inhibit the activity of GPX4. Although NTP + RSL3 had no significant effect on GPX4 activity when compared with the treatment with NTP only (Fig. 4A), the expression of GPX4 protein increased significantly after treatment with NTP + RSL3 (Fig. 4B). We reasoned that the induced GPX4 expression after NTP + RSL3 treatment might represent an adaptive response wherein cancer cells tried to recover cell survival in response to excessive ROS.

However, the expression of xCT, a transporter of cystine, distinctly decreased after NTP + RSL3 treatment (Fig. 4B). The significantly increased ROS and decreased GSH (Fig. 4C) also indicated the dysfunction of xCT after NTP + RSL3 treatment. Furthermore, treatment with β-mercaptoethanol, acting as a carrier for taking cystine into cells to promote more intracellular GSH synthesis in the absence of xCT, effectively rescued the cell viability (Fig. 4D), showing the dysfunction of xCT after NTP + RSL3 treatment. To further verify the role of xCT, we overexpressed it in Calu-1 cells (Fig. 4E). As expected, xCT overexpression rescued the reduced viability of NTP + RSL3-treated cells (Fig. 4F). These results suggest that NTP + RSL3 treatment exhibited combined lethal effect by synergistically inhibiting xCT expression and inducing ferroptosis in NSCLC cells.

### NTP + RSL3 treatment promoted xCT lysosomal degradation via ROS/AMPK/mTOR axis

**Fig. 5:**
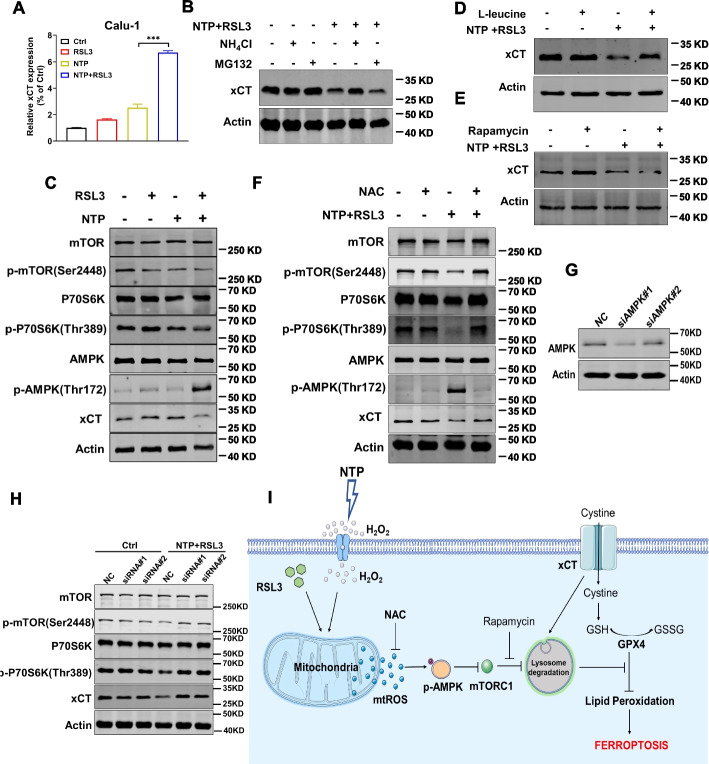
NTP + RSL3 treatment promotes xCT lysosomal degradation via ROS/AMPK/mTOR axis. **A** Transcription level of xCT. **B** Effect of NH_4_Cl (20 mM) or MG132 (5 μM) on the expression of xCT protein. **C** The protein level of mTORC1 pathway and phosphorylation of AMPK after NTP + RSL3 treatment. **D** Effect of L-leucine (20 mM) on the protein expression of xCT. **E** Effect of rapamycin (100 μM) on the protein expression of xCT. **F** Effect of NAC (5 mM) on the expression of mTORC1, AMPK phosphorylation and xCT expression. **G** The interference effect of siRNA on AMPK expression in Calu-1 cells. **H** The levels of mTORC1 phosphorylation and xCT expression after NTP + RSL3 with siRNA treatment in Calu-1 cells. **I** Schematic overview of mechanism underlying the ferroptosis induction by NTP + RSL3 via ROS/AMPK/mTOR axis promoting xCT lysosomal degradation

As an important transporter protein located on the cytomembrane, xCT is refractory to be degraded relatively [37]. The results in Fig. 5A showed that NTP + RSL3 treatment increased the transcription of xCT but decreased the protein level of xCT distinctly (Fig. 4C, Fig 5A). To explore the regulation of degradation, we treated the cells with proteasome inhibitor MG132 or lysosome inhibitor NH_4_Cl. Interestingly, NH_4_Cl efficiently elevated xCT expression to the control level while MG132 showed no distinct effect after NTP + RSL3 treatment (Fig. 5B). These results indicate that the decreased xCT protein after NTP + RSL3 treatment should be degraded in a lysosome-dependent manner.

mTORC1 is an important protein complex in charge of numerous cellular processes involved in cell growth and metabolism, such as lipid metabolism and autophagy [38]. Hang et al. found that cystine starvation reduced mTOR localization on lysosomes and suppressed mTORC1 activation [39], while Conlon et al. demonstrated that mTOR inhibition blocked mTORC1-mediated protein synthesis and regulated ferroptosis resistance under cysteine deprivation [40]. We hypothesized that mTORC1 modulated xCT expression via lysosomal degradation. Our results confirmed activation of AMPK and inhibition of mTORC1 pathway after treatment (Fig. 5C). Furthermore, we investigated the possible role of mTORC1 in regulating xCT degradation by treating the cells with L-leucine, a specific activator of mTORC1, or its inhibitor rapamycin. The results showed that L-leucine treatment elevated the xCT protein level (Fig. 5D) while rapamycin treatment decreased the xCT expression after NTP + RSL3 treatment (Fig. 5E), which proved that mTORC1 played a key role in xCT degradation regulation.

Considering that excessive ROS acted as the executor for the combined lethal effect, we demonstrated the initiating role of ROS to the downstream pathways by NAC treatment, and the results (Fig. 5F) showed that NAC treatment effectively eliminated AMPK phosphorylation, mTORC1 inhibition and xCT degradation. These results suggest the excessive ROS induced by NTP + RSL3 treatment should be the stimulant to activation of the AMPK pathway. To further confirm the AMPK/mTOR axis, we interfered AMPK expression with the specific siRNA and the result showed the inhibition of mTORC1 was relieved and the mTORC1 signal pathway, including the phosphorylation levels of mTOR and p70S6 kinase, was recovered to the control level after NTP + RSL3 treatment (Fig. 5G, H). Taken together, our results indicate that ROS activated the AMPK pathway to suppress mTORC1 function, promoted xCT lysosomal degradation and ultimately caused ferroptosis.

### PAM+RSL3 treatment synergistically suppressed tumor growth in vivo

**Fig. 6:**
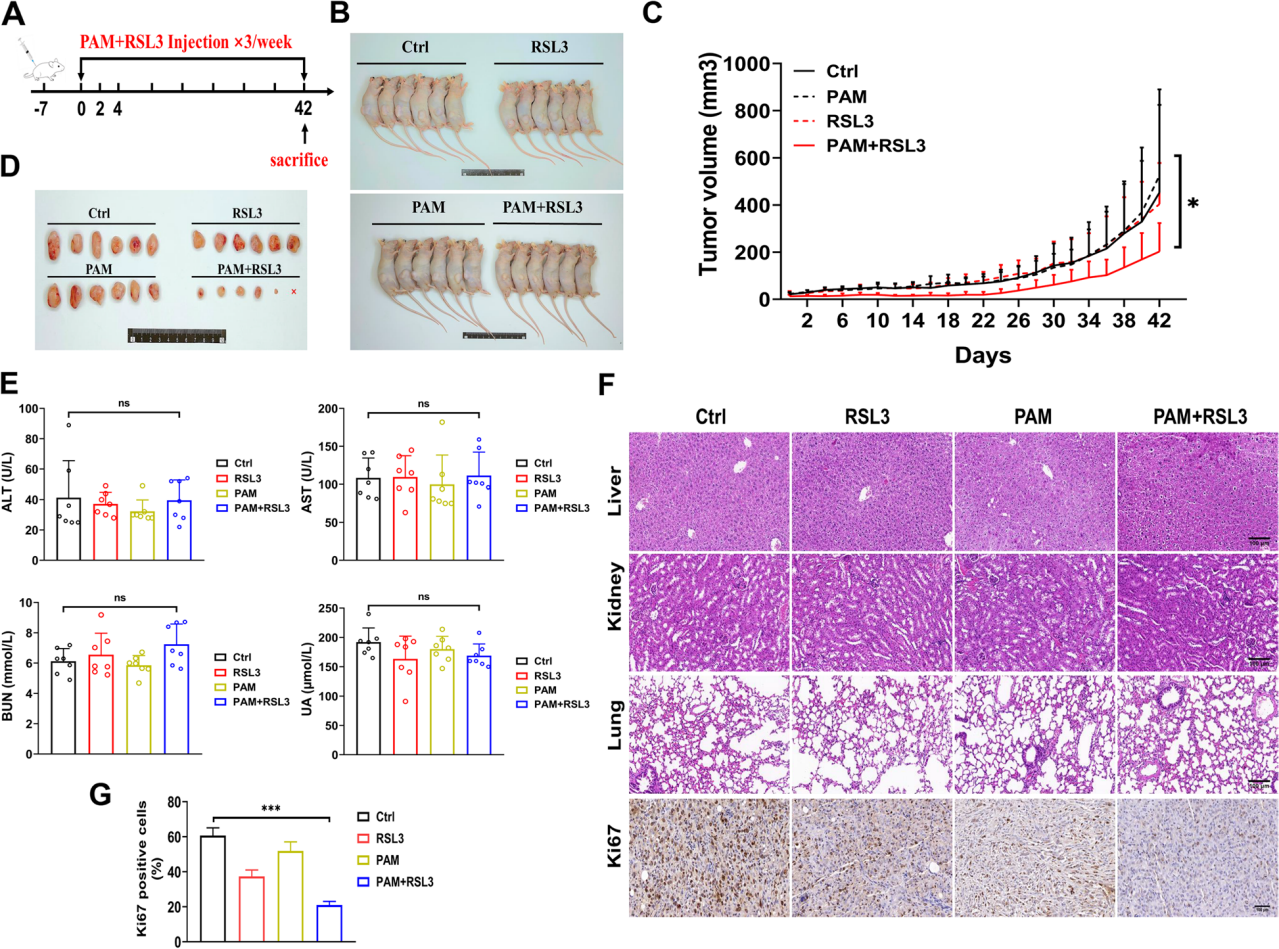
PAM+RSL3 treatment suppresses tumor growth in vivo. **A** Mice treatment schedule. **B** The general images of mice on Day 42. **C** Tumor growth curve. **D** The images of excised tumors. **E** Level of ALT, AST, BUN and UA in the mice blood. **F** Representative HE images of liver, kidney, lung tissues of mice and IHC images of Ki 67. Scale bar, 100 μm. **G** Qualification of Ki67 in F. *: *p* < 0.05

As shown in Fig. 6A, the Calu-1 xenografts burdened on the nude mice were treated with PAM and RSL3 for three times per week. After six weeks, the mice were sacrificed, and the subcutaneous tumors and interested organs were isolated. Compared with the mice treated with only PAM or RSL3, PAM+RSL3 treatment significantly suppressed tumor growth (Fig. 6B-D). Moreover, the results of Ki 67 immunohistochemistry showed the proliferation of tumor cells was also efficiently inhibited by the combined treatment (Fig. 6F&G).

Considering the potential cytotoxicity of RSL3 to normal tissues, we detected alanine aminotransferase (ALT), aspartate aminotransferase (AST), blood urea nitrogen (BUN) and urine acid (UA) in the blood samples and the pathological changes of liver, kidney and lung tissues. No distinct differences were observed between the PAM+RSL3 and control groups (Fig. 6E&F). Collectively, these results suggest that PAM+RSL3 treatment synergistically suppressed tumor growth in vivo without significant toxicity to normal tissues.

## Discussion

As one of the classical GPX4 inhibitors, RSL3 is selectively lethal to oncogenic RAS mutant cell lines and widely used in research in vitro. Nonetheless, the poor pharmacokinetic properties have limited its systemic usage in vivo while high doses of RSL3 (≥100 mg/kg) have caused potential hepatotoxicity [20]. Some strategies, e.g., by combining RSL3 with other clinical drugs, have been proposed for better curative efficacy. For example, combining cetuximab with RSL3 induced ferroptosis via suppressing the Nrf2/HO-1 signaling pathway in KRAS mutant colorectal cancer cells [41]. Moreover, Sorafenib combined with RSL3 synergistically improved the anticancer efficacy in hepatocellular carcinoma cells [42]. In this study, we found that NTP could also cooperate with RSL3 to induce ferroptosis rapidly in NSCLC cells in vitro and in vivo but no distinct toxicity to normal lung cells and tissues was detected (Fig. 1, Fig. 6 E, F). The synergism of NTP and low-concentration RSL3 contributed to enhance the availability of RSL3 in inducing ferroptosis in NSCLC while avoiding its underlying toxicity. Our data supported that NTP had strong potential to collaborate with clinical drugs for making available more curative targets and getting better therapeutic efficacy.

In the past decade, NTP have been shown the potential to induce various cell death pathways, including apoptosis [43], autophagy [12], senescence [44] and pyroptosis [14]. Choi et al. confirmed that NTP induced apoptosis in lung cancer cells by causing DNA damages and cell cycle arrest [5]. Our previous study proved that the ROS generated by NTP could effectively initiate GSDME-dependent pyroptosis in lung cancer cells [14], showing the potential of NTP in lung cancer treatment. In the present study, combination of low-dose NTP and low-concentration RSL3 significantly boosted lipid peroxidation accumulation (Fig. 2B, C), and gave rise to mitochondrial damages (Fig. 2D), while NTP or RSL3 treatment alone showed little killing effect. Our data revealed that NTP could improve the ferroptosis sensitivity and RSL3 availability in tumor cells, providing new strategies to lung clinical cancer treatment. Furthermore, low dose NTP may also have influence in the sensitivity of other cell death pathways and have immense potential in combining with other chemical drugs for curing cancer.

A previous study showed that NTP could generate numerous ROS/RNS, which determined the multiple biological effects of NTP, in the liquid phase. NO^2−^ induced by NTP could play a notable role in re-sensitizing tumors to chemotherapy or radiation therapy [45]. ONOO^−^ and H_2_O_2_ were also crucial for inducing apoptosis in PAM [26], [41], [46]. Our data showed that NTP combined with RSL3 could substantially heighten the intracellular ROS level (Fig. 3A) and H_2_O_2_ was a key active ingredient in PAM which caused the lethal effect (Fig. 3C). A supplement of H_2_O_2_ in a similar concentration was applied to mimic NTP treatment and similar changes in cell viability, lipid peroxidation and ROS level were observed (Fig. 3F-J), proving the H_2_O_2_-dependent cell killing effect of NTP treatment. Although we verified H_2_O_2_ as a critical ingredient in PAM to promote ferroptosis, the role of other active species in cell death induction could not be eliminated and further studies are still needed.

As an essential antiport protein on the cell membrane, xCT regulates cellular cysteine and glutathione homeostasis. The proteins of p53 and Nrf2 were the main factors in regulating xCT expression at the transcription level [47, 48]. Although several reports have revealed xCT degradation in proteasome-dependent manner [17], [49], lysosome was also important in regulating xCT depredation [50, 51]. In accordance with these studies, our results displayed that NTP combined with RSL3 promoted xCT degradation by lysosome rather than transcriptional regulation or proteasome-mediated protein degradation (Fig. 5A, B). Increased intracellular ROS stimulated the AMPK signaling pathway, which sensed energy balance and cell stress, and then suppressed mTORC1 function, finally resulting in xCT degradation in lysosome (Fig. 5C, D, E).

## Conclusion

In conclusion, our results revealed the lethal effect of combination of NTP with RSL3 in triggering ferroptosis rapidly. We demonstrated mitochondrial ROS induced ferroptosis in combination treatment and H_2_O_2_ played a vital role in killing cancer cells. Mechanistically, NTP + RSL3 promoted xCT lysosomal degradation via ROS/AMPK/mTOR axis. Taken together, our findings proved the synergism of NTP and RSL3, revealed the possibilities of NTP in combination with clinical drugs for better beneficial effects, and provided theoretical basis and potential strategy for clinical lung cancer treatment.

## Data Availability

Data will be made available on request.

## Abbreviations

NTP: Non-thermal plasma
RSL3: (1S, 3R)-RSL3
ROS: Reactive oxygen species
RNS: Reactive nitrogen species
Nec-1 s: Necrosis-1 s
CQ: Chloroquine
Fer-1: Ferrostatin-1
NAC: N-acetyl-L-cysteine
PAM: Plasma-activated medium
NSCLC: Non-small cell lung cancer
ALT: Alanine aminotransferase
AST: Aspartate aminotransferase
BUN: Blood urea nitrogen
UA: Urine acid
